# Cytogenetic characterization of the malignant primitive neuroectodermal SK-PN-DW tumor cell line

**DOI:** 10.1186/s12885-019-5625-1

**Published:** 2019-05-02

**Authors:** Na Du, Wanguo Bao, Kaiyu Zhang, Xianglan Lu, Rebecca Crew, Xianfu Wang, Guangming Liu, Feng Wang

**Affiliations:** 1 Department of Infectious Diseases, the First Hospital of Jilin University, 71 Xinmin Street, Changchun, Jilin, 130021 People’s Republic of China; 2 Department of Pediatrics, University of Oklahoma Health Sciences Center, Oklahoma City, OK 73104 USA; 3 Department of Gastroenterology, the First Hospital of Jilin University, Changchun, Jilin, 130021 People’s Republic of China

**Keywords:** SK-PN-DW, Primitive neuroectodermal tumor, PNET, Ewing sarcoma

## Abstract

**Background:**

The SK-PN-DW cell line was established in 1979 and is commercially available. Despite the use of this cell line as an in vitro model for functional and therapeutic studies of malignant primitive neuroectodermal tumor (PNET), there is a lack of complete information about the genetic alterations that are present at the cytogenetic level. Thus, the current study aimed to characterize the cytogenetic profile of this cell line.

**Methods:**

Routine G-banded chromosome analysis, fluorescence in situ hybridization, and oligonucleotide array comparative genomic hybridization assays were performed to characterize the chromosomal changes in this cell line.

**Results:**

The G-banded karyotype analysis showed that the number of chromosomes in this cell line ranged between 36 and 41. Importantly, all cells displayed a loss of chromosomes Y, 11, 13, and 18. However, some cells showed an additional loss of chromosome 10. Additionally, the observed structural changes indicated: a) unbalanced translocation between chromosomes 1 and 7; b) translocation between chromosomes 11 and 22 at breakpoints 11q24 and 22q12, which is a classical translocation that is associated with Ewing sarcoma; c) a derivative chromosome due to a whole arm translocation between chromosomes 16 and 17 at likely breakpoints 16p10 and 17q10; and d) possible rearrangement in the short arm of chromosome 18. Moreover, a variable number of double minutes were also observed in each metaphase cell. Furthermore, the microarray assay results not only demonstrated genomic-wide chromosomal imbalance in this cell line and precisely placed chromosomal breakpoints on unbalanced, rearranged chromosomes, but also revealed information about subtle chromosomal changes and the chromosomal origin of double minutes. Finally, the fluorescence in situ hybridization assay confirmed the findings of the routine cytogenetic analysis and microarrays.

**Conclusion:**

The accurate determination of the cytogenetic profile of the SK-PN-DW cell line is helpful in enabling the research community to utilize this cell line for future identity and comparability studies, in addition to demonstrating the utility of the complete cytogenetic profile, as a public resource.

## Background

Typically, cell lines play a fundamental role in biomedical research, where they are used as in vitro models through which to investigate the mechanisms of disease initiation and progression, drug efficacy and therapeutic outcomes. In addition, they appear to be important in the study of rare or atypical cancers, where primary biological specimens are difficult to obtain. Thus, the importance of results obtained using cell lines is completely dependent upon their reliability and authenticity. In this regard, for decades, the misidentification of cell lines has been a major and significant concern in the scientific community, and significant efforts have only recently been made to address this issue on a large scale [1, 2]. Currently, several funding agencies and publications require a statement or proof of the authenticity of the cell lines that are used in the specific study before even considering them for further review. In this background, the cell repositories and creators of cell lines usually perform authentication studies. However, there is still a possibility of the drifting of cell lines due to various factors, including cells obtained from secondary sources, chromosomal instability, continuous culturing and sub-culturing, or culturing in areas that are exposed to other contaminating cell lines or mycoplasma.

The initial authentication of any new cell line involves performing a panel of tests that were designed to address issues of inter- and intra-species contamination, tissue of origin, mycoplasma or other microbial contaminants, and genetic stability. However, re-authentication of a cell line after it is received in the laboratory, or prior to its use, has been simplified to a few tests. One of the most common methods used for re-authentication is SRT profiling, also known as DNA fingerprinting. This method is relatively fast and inexpensive. However, it is not able to detect numerical changes or marker chromosomes, and thus has a limited capacity in the analysis of mixed cell populations. Solid tumor cell lines often display complex genetic arrangements, including multiple numerical and structural aberrations with significant variation among different cells of the same tumor [3]. Thus, cytogenetic analysis by a well-trained individual seems to be the best method, with the highest sensitivity and versatility, through which to characterize the chromosomal changes of a cell line. Thus, it would be sufficient to say that establishing the authenticity of any cell line would require a true cytogenetic profile comparison. Unfortunately, the majority of the cytogenetic analyses of many cell lines was performed in the late 1980s and 1990s, when techniques were significantly less sensitive and not very robust.

Primitive neuroectodermal tumors (PNETs) are aggressive, highly malignant and small round cell tumors with diverse clinical manifestations. These PNETs are more (4.6 times) likely to arise in children and adolescents, with an incidence rate of 0.62 per million people in the United States. Therefore, PNETs are classified as a rare cancer [4]. PNETs are usually classified into three types, based on the tissue of origin: a) CNS, arising from the central nervous system; b) neuroblastoma, arising from the autonomic nervous system; and c) peripheral, arising from any other tissue [5]. Currently, the diagnosis of a PNET is based on MRI and CT imaging; however, since these tumors can arise from a vast variety of tissues, cytogenetic confirmation of then tumor biopsy is quite essential [6, 7]. Notably, peripheral PNETs (or pPNETs) have been shown to belong to the Ewing family of tumors, which are diagnosed by the presence of the t (11:22) chromosomal variation, characteristic of the tumors in this family [8]. However, due to the low incidence rate of this tumor type, cell lines play a prominent role in their scientific research, as primary tumor specimens are very hard to find. The commercially available SK-PN-DW cell line consists of immortalized pPNET cells derived from the presacrum of a 17-year-old male in 1978. This cell line was established by C Helson in 1979, and was initially characterized by conventional G-banding [9]. Since then, this cell line become an very important tool for PNETs, especially for the study of tumorigenesis mechanisms and the development of anti-tumor drugs [10, 11], but very few studies have further analyzed the genetic profile of this cell line.

In our current study, we analyzed the SK-PN-DW cell line and define the common chromosomal numerical and structural changes, using modern technology, with the intention of providing the comprehensive cytogenetic profile of this cell line as a public resource for the research community who are using this cell line to further study PNET biology. At the same time, we validated a hypothesis that the cell line undergoes structural changes after passage, which may affect its function. This hypothesis requires a large number of follow-up experiments to prove.

## Methods

### Cell line and cell culture

The primitive neuroectodermal cell line, SK-PN-DW, was obtained from the American Type Culture Collection (ATCC, Manassas, VA, USA; Lot# 2056389) in 2011, and was grown in RPMI 1640 medium (Corning) supplemented with 12% fetal bovine serum (FBS; Gibco), 1 x Penicillin-Streptomycin (Gibco), and 2 mM-glutamine (Gibco), at 37 °C and 5% CO_2_ in an incubator. Later, the cells were frozen for subsequent studies.

### G-banding and karyotype analysis

The cells were collected, in the metaphase stage, by exposing them to colcemid solution (0.05 μg/ml; Gibco) for one hour. The cells were then harvested from the surface of the culture flask through a brief incubation with 0.05% trypsin-EDTA (GIBCO). Next, the harvested cells were treated with 0.075 M KCl hypotonic solution, and then fixed through three incubations with Carnoy’s fixative (3:1 methanol to acetic acid) before being placed on to glass slides. The slides were then incubated at 58 °C for 16 h before staining.

G-banding was achieved through a brief exposure of the cells to 0.1% trypsin (w/v) DPBS solution, followed by two rinses with 0.9 M NaCl solution, and subsequent staining with Giemsa stain (EMD). The final images were captured and analyzed using CytoVision software version 7 (Applied Spectral Imaging, Santa Clara, CA, USA).

### Fluorescence in situ hybridization (FISH) analyses

FISH analyses were performed using multiple DNA probes that were purchased from Abbott Molecular (Des Plaines, IL, USA) and used based on manufacture’s protocols, with minor changes. The whole chromosome painting probes were used for chromosomes 1, 7, 8, 16, 17, 18, 21, and 22 analysis, while centromere probes were used for chromosomes X, Y, 3, 10, 11, 16, 17 and 18 analysis. Locus-specific probes that were designed for the genes EGR1 on 5q31, cMYC on 8q24, IGH1 on 14q32, and EWSR1 on 22q12, in addition to Vysis probe sets LSI 13 on 13q14, and LSI21 on 21q22,13-q22.2 were used. Overall, a total of 200 interphase cells, and 20 metaphase cells were analyzed with each probe. The digital images of specific hybridization signals were processed using CytoVision software version 7 (Applied Spectral Imaging, Santa Clara, CA, USA).

### Array comparative genomic hybridization

Array comparative genomic hybridization (CGH) was performed, as has been described previously [12]. Briefly, the reference DNA was purchased from Agilent (Agilent Corporation, Santa Clara, CA, USA), while the cell line DNA was labeled with either cyanine 3 (Cy-3) or cyanine 5 (Cy-5) by random priming, according to manufacturer’s instructions. Equal amounts of reference and cell line DNA were mixed, and then loaded onto an Agilent 2 × 400 K oligo microarray chip (Agilent Technologies, Santa Clara, CA, USA). The hybridization was performed for 40 h at a temperature of 67 °C. Slides were then washed and scanned using a NimbleGen MS 200 Microarray Scanner (NimbleGen System Inc., Madison, WI, USA). The data were analyzed using Agilent’s CytoGenomics 2.7 software (Agilent Technologies, Santa Clara, CA, USA).

Finally, the chromosomal anomalies that were detected through routine G-banded chromosomal analysis, FISH, and array CGH were described based on the guidelines in “An International System for Human Cytogenetic Nomenclature (2013)”.

## Results

### Routine G-banded chromosomal karyotyping

**Fig. 1:**
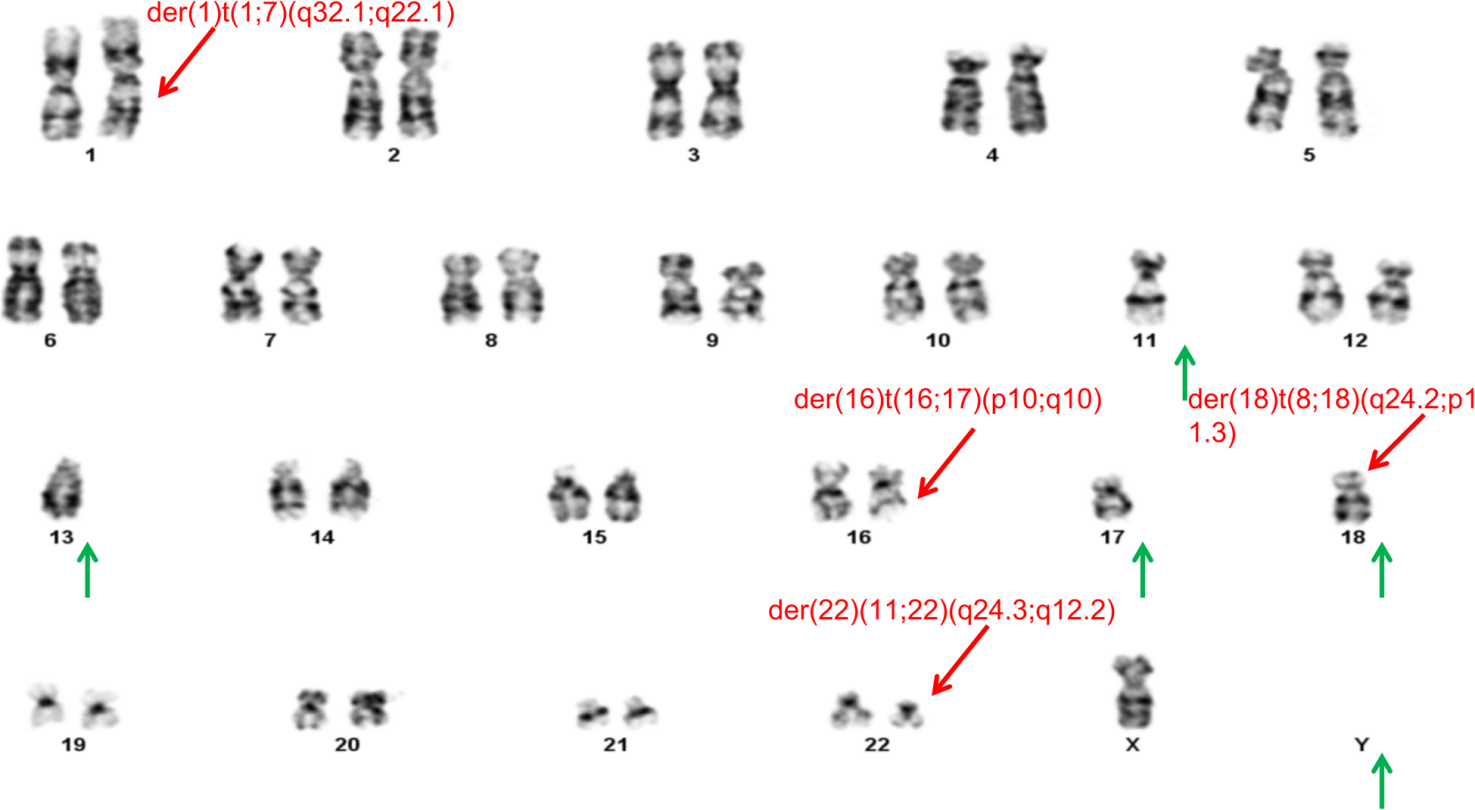
A representative abnormal karyotype showing chromosomal structural and numerical changes in the SK-PN-DW cell line: Arrows indicate the following rearrangements; der(1)t(1;7)(q32.1;q22.1), der(16)t(16;17)(p10;q10), der [14](8;18)t(q24.2;p11.3), der(22)t(11;22)(q24.3;q12.2), 4–60 double minutes (indicated by red arrows), and loss of Y chromosome and chromosomes 11, 13, 17, and 18 (indicated by green arrows)

**Fig. 2:**
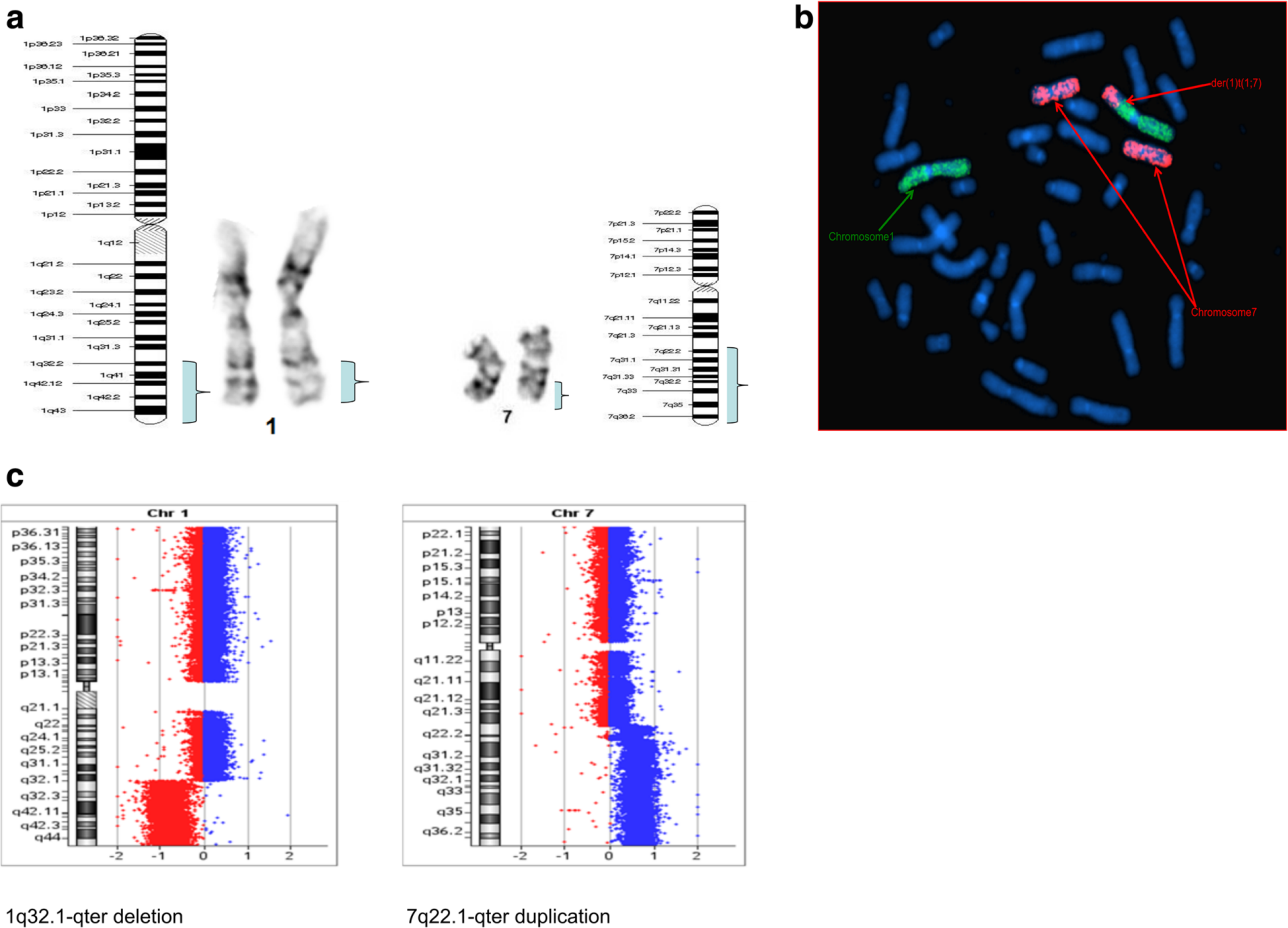
1) Whole chromosome 1 and 7 painting probes showing part of chromosome 7q was present on derivative chromosome 1, due to translocation: der(1)t(1;7)(q32.1;q22.1). 2) Imaging shows that there is partial overlap of the labeling for chromosome 1 (green) and chromosome 7/der(1)t(1;7) (red). 3) The CGH array indicating partial gain of chromosome 7 at q22.1(blue bar), and partial loss of chromosome 1 at q32.1(red bar)

**Fig. 3:**
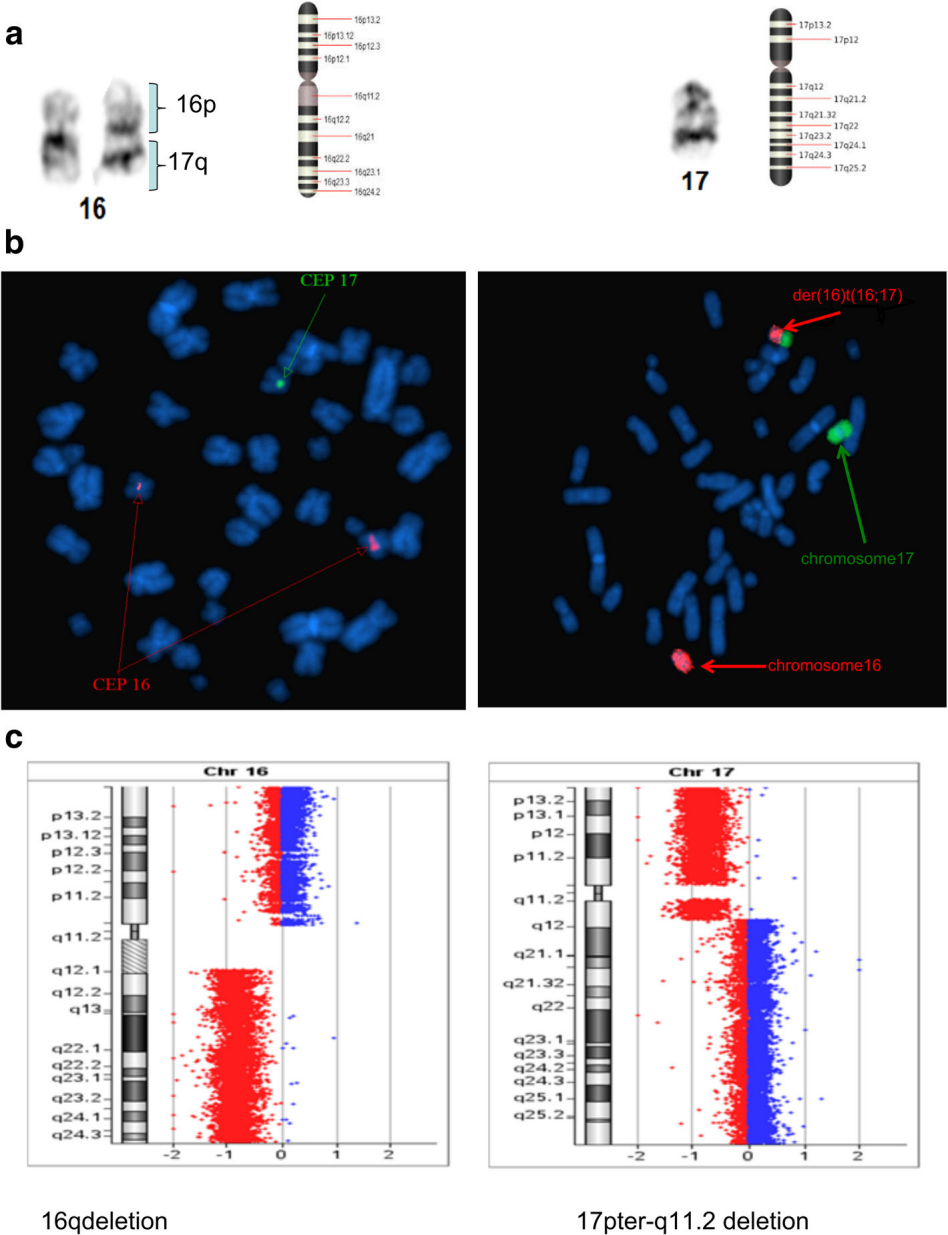
1) Whole chromosome 16 and 17 painting probes showing translocation der(16)t(16;17)(p10;q10) between chromosome 16 and 17. 2) Images showing the CEP 16 probe (red) and the CEP 17 probe (green). 3) CGH array showing loss of entire q and p arms of chromosomes 16 and 17

**Fig. 4:**
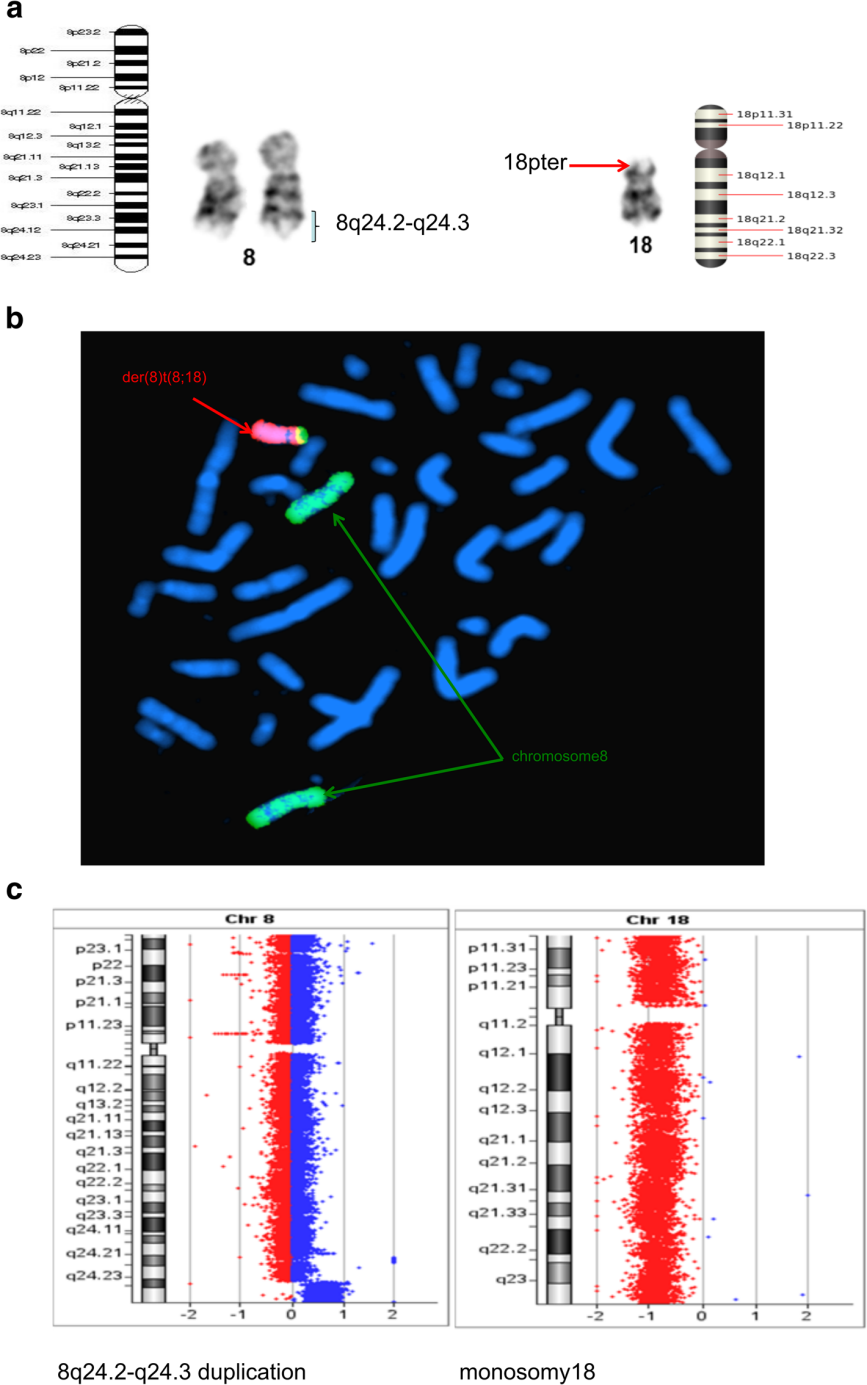
1) Whole chromosome 8 and 18 painting probes showing translocation der(18)t(8;18)(q24.2p11.3). 2) Images showing partial overlap of labeled chromosome 18 (red) and chromosome 8 (green) confirming translocation. 3) CGH array analysis showing loss of complete chromosomes 18 (red bar) and high-level gain at 8q24 (blue bar) region

**Fig. 5:**
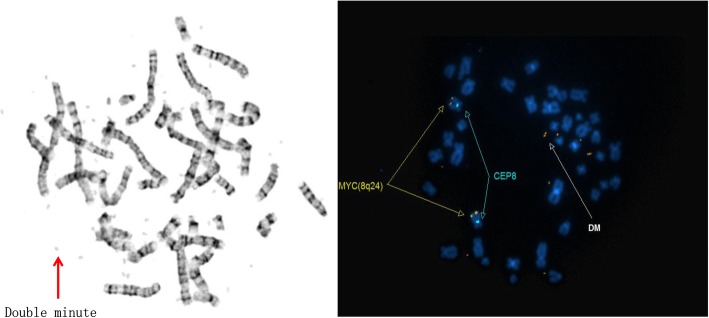
CEP 8 and cMYC 8q24 specific probes confirm the presence of cMYC sequences within the observed double minutes (DM; as indicated by the red arrow)

**Fig. 6:**
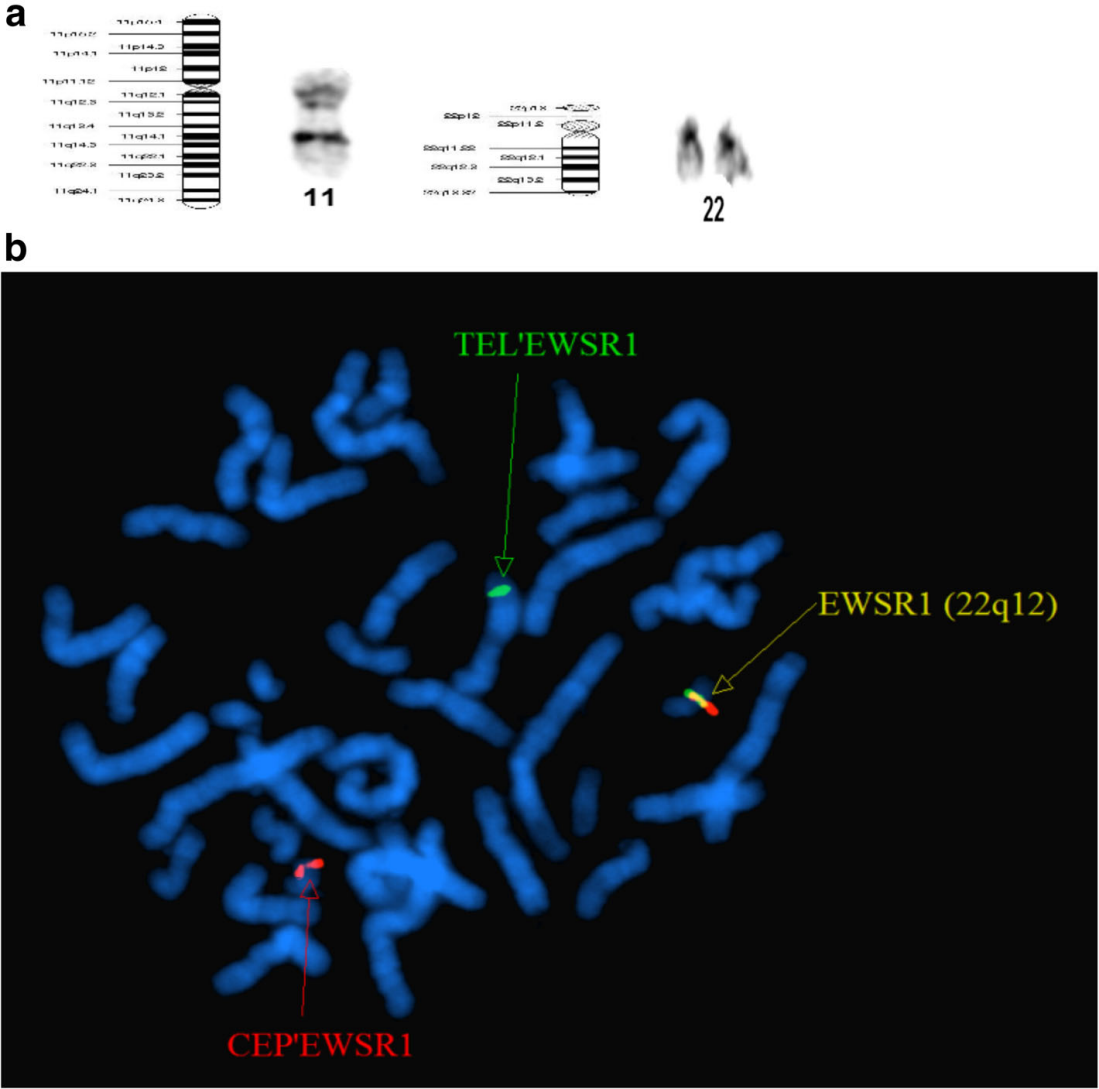
1) Whole chromosome 11 and 22 painting probes. 2) Fusion-break part EWSR1 specific probe labeled chromosome 22q12 (orange), while chromosome 22 (red) and 11 (green) show translocation, der(11)t(11;22)(q24.3;q12.2)

In total, 20 cells at the metaphase stage were analyzed. All analyzed cells displayed consistent chromosomal anomalies with a modal number of chromosomes ranging from 36 to 41. (Table 1) The numerical abnormalities included the loss of the Y chromosome, monosomy of chromosomes 11, 13, 17 and 18, and mosaic monosomy of chromosome 10. In addition, double minutes (DMs) were also observed in all cells, ranging in quantity from 4 to 60. Importantly, the classical translocation associated with Ewing sarcoma was also observed between chromosomes 11 and 22 at 11q24 and 22q12 breakpoints. Other structural chromosome changes included unbalanced translocation between the terminal q arms of chromosomes 1 and 7, a derivative chromosome arising from whole arm translocation between chromosomes 16 and 17 at the likely breakpoints 16p10 and 17q10, and the possible rearrangement of the short arm of chromosome 18, (Figs. 1, 2a, 3a, 4a, 5 and 6a).

**Table 1:** The karyotype results of 20 metaphase cells on SK-PN-DW cell line

No.	Karyotype results
Cell-1	40X, −Y, der(1)t(1;7)(q32.1;q22.1), − 11, t(11;22)(q24;q12), − 13, der(16;17)(p10;q10), − 18, add [14](p11.2), − 19
Cell-2	40X, −Y, der(1)t(1;7)(q32.1;q22.1), − 10, − 11, t(11;22)(q24;q12), − 13, der(16;17)(p10;q10), − 18, add [14](p11.2)
Cell-3	36X, −Y, der(1)t(1;7)(q32.1;q22.1), − 1, − 2, − 3, − 4, − 9, − 11, t(11;22)(q24;q12), − 13, der(16;17)(p10;q10), − 18, add [14](p11.2)
Cell-4	40X, −Y, der(1)t(1;7)(q32.1;q22.1), − 10, − 11, t(11;22)(q24;q12), − 13, der(16;17)(p10;q10), − 18, add [14](p11.2)
Cell-5	41X, −Y, der(1)t(1;7)(q32.1;q22.1), − 11, t(11;22)(q24;q12), − 13, der(16;17)(p10;q10), − 18, add [14](p11.2), − 21
Cell-6	41X, −Y, der(1)t(1;7)(q32.1;q22.1), − 11, t(11;22)(q24;q12), − 13, − 16, − 18, add [14](p11.2)
Cell-7	40X, −Y, der(1)t(1;7)(q32.1;q22.1), − 10, − 11, t(11;22)(q24;q12), − 13, − 16, − 18, add [14](p11.2)
Cell-8	40X, −Y, der(1)t(1;7)(q32.1;q22.1), − 10, − 11, t(11;22)(q24;q12), − 13, der(16;17)(p10;q10), − 18, add [14](p11.2)
Cell−9	39X, −Y, der(1)t(1;7)(q32.1;q22.1), − 10, − 11, t(11;22)(q24;q12), − 13, der(16;17)(p10;q10), − 18, add [14](p11.2), − 20
Cell-10	39X, −Y, der(1)t(1;7)(q32.1;q22.1), − 10, − 11, t(11;22)(q24;q12), − 13, der(16;17)(p10;q10), − 18, add [14](p11.2), − 20
Cell-11	41X, −Y, der(1)t(1;7)(q32.1;q22.1), − 11, t(11;22)(q24;q12), − 13, der(16;17)(p10;q10), − 18, add [14](p11.2)
Cell-12	40X, −Y, der(1)t(1;7)(q32.1;q22.1), − 10, − 11, t(11;22)(q24;q12), − 13, der(16;17)(p10;q10), − 18, add [14](p11.2)
Cell-13	40X, −Y, der(1)t(1;7)(q32.1;q22.1), − 10, − 11, t(11;22)(q24;q12), − 13, der(16;17)(p10;q10), − 18, add [14](p11.2)
Cell-14	40X, −Y, der(1)t(1;7)(q32.1;q22.1), − 7, − 11, t(11;22)(q24;q12), − 13, der(16;17)(p10;q10), − 18, add [14](p11.2)
Cell-15	40X, −Y, der(1)t(1;7)(q32.1;q22.1), − 11, t(11;22)(q24;q12), − 13, der(16;17)(p10;q10), − 18, add [14](p11.2)
Cell-16	40X, −Y, der(1)t(1;7)(q32.1;q22.1), − 10, − 11, t(11;22)(q24;q12), − 13, der(16;17)(p10;q10), − 18, add [14](p11.2)
Cell-17	41X, −Y, der(1)t(1;7)(q32.1;q22.1), − 11, t(11;22)(q24;q12), − 13, der(16;17)(p10;q10), − 18, add [14](p11.2)
Cell-18	40X, −Y, der(1)t(1;7)(q32.1;q22.1), − 10, − 11, t(11;22)(q24;q12), − 13, der(16;17)(p10;q10), − 18, add [14](p11.2)
Cell-19	40X, −Y, der(1)t(1;7)(q32.1;q22.1), − 11, − 13, der(16;17)(p10;q10), − 18, add [14](p11.2), − 19
Cell-20	41X, −Y, der(1)t(1;7)(q32.1;q22.1), − 11, t(11;22)(q24;q12), − 13, der(16;17)(p10;q10), − 18

### Fluorescence in situ hybridization

Furthermore, conventional two-color FISH analysis was performed using arm-specific probes for chromosomes 13, 18, 21, X and Y. This analysis confirmed monosomy at chromosomes 13, 18 and Y in all cells. The CEP (chromosome enumeration probe) 10 and 11 confirmed monosomy 11 in all cells, while mosaic monosomy 10 in approximately 11.5% of the cells (23/200). The karyotype and array CGH results indicated that there were rearrangements between chromosomes 1 and 7; 8 and 18; 11 and 22; and 16 and 17. Because of this, whole chromosome painting probes were used for chromosome pairs 1 and 7. This confirmed the translocation of chromosome 7 material to the terminal q arm of chromosome 1 (Fig. 2b). In addition, the CEP 16 and 17 probes confirmed monosomy 17 in all cells, and revealed the presence of derivative chromosome 16 consisting of the p arm of chromosome 16 and q arm of chromosome 17 (Fig. 3b). Moreover, the whole chromosome painting probes for chromosomes 8 and 18 also confirmed the translocation of chromosome 8 material to the terminal p arm of chromosome 18 (Fig. 4b). In addition, the CEP 8 and cMYC 8q24 specific probes were used to confirm cMYC sequences within the observed DMs (Fig. 5). Finally, EWSR1 22q12 gene breakpoint probe also verified the translocation between chromosome 11 and 22 at (11q; 22q) regions (Fig. 6b). Overall, the cumulative results have been summarized in Figs. 2 (a-c), 3 (a-c), 4 (a-c), 5, 6 (a-b). Additionally, we used FISH assay with multiple combinations of FISH probes, including whole chromosome painting probes for chromosomes 1, 7, 8, 16, 17, and 18, and arm-specific probes for chromosomes 13, 16, 17, 18, 21, 22, X, and Y. Overall, the following indications were observed; der [1],t(1;7)(1q;7q); der [11],t(11;22) (11q;22q); der [13], t(16;17) (16q;17q); der [14],t(8;18) (8q;18p).

### Comparative genomic hybridization

**Fig. 7:**
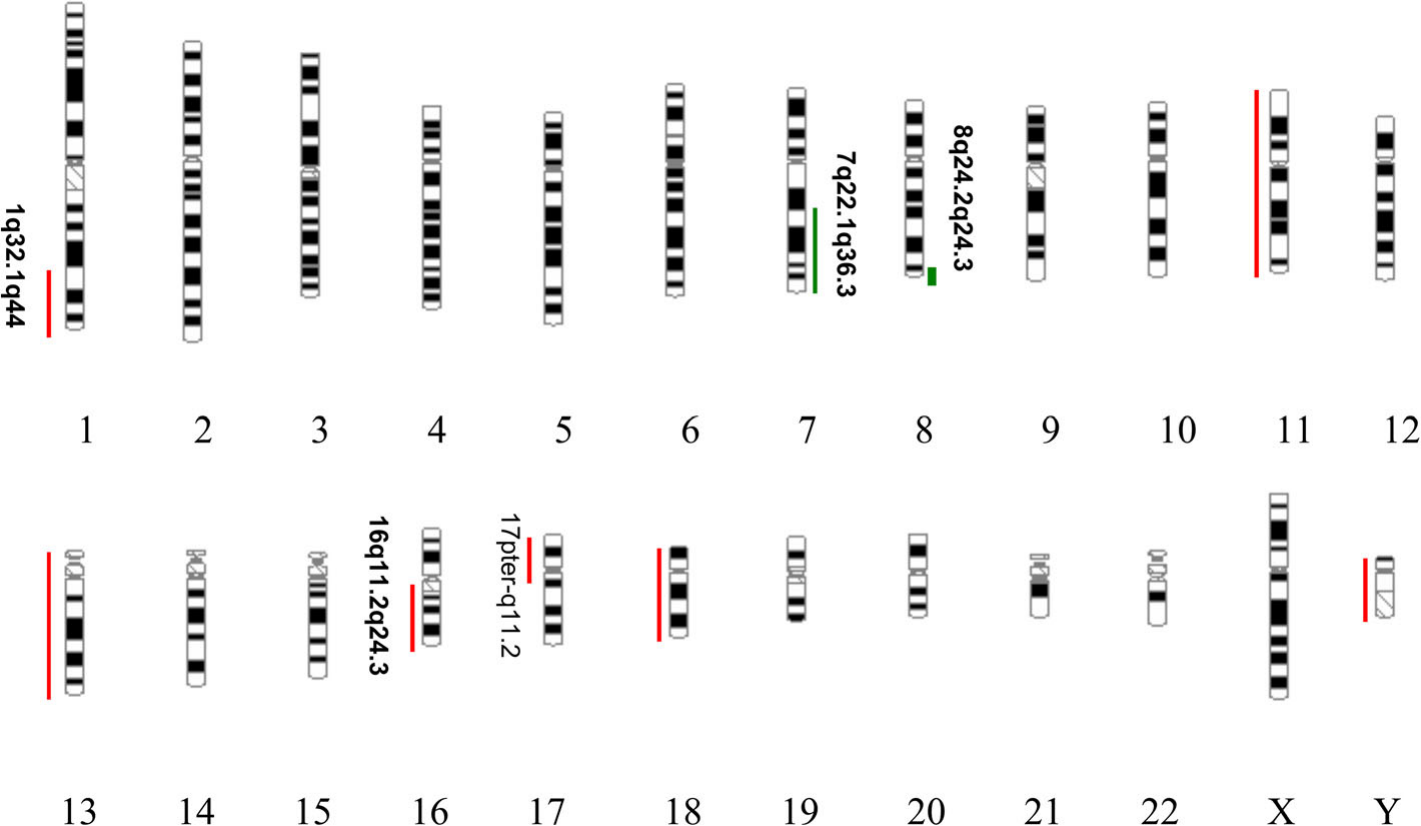
Ideogram summarizing the chromosomal imbalances detected by array CGH. Vertical red lines to the left of the chromosome ideogram indicate chromosomal loss. The thin vertical green line to the right of the chromosome ideogram indicates chromosomal gain, while the heavy green line to the right of the chromosome ideogram indicates segmental amplification

**Fig. 8:**
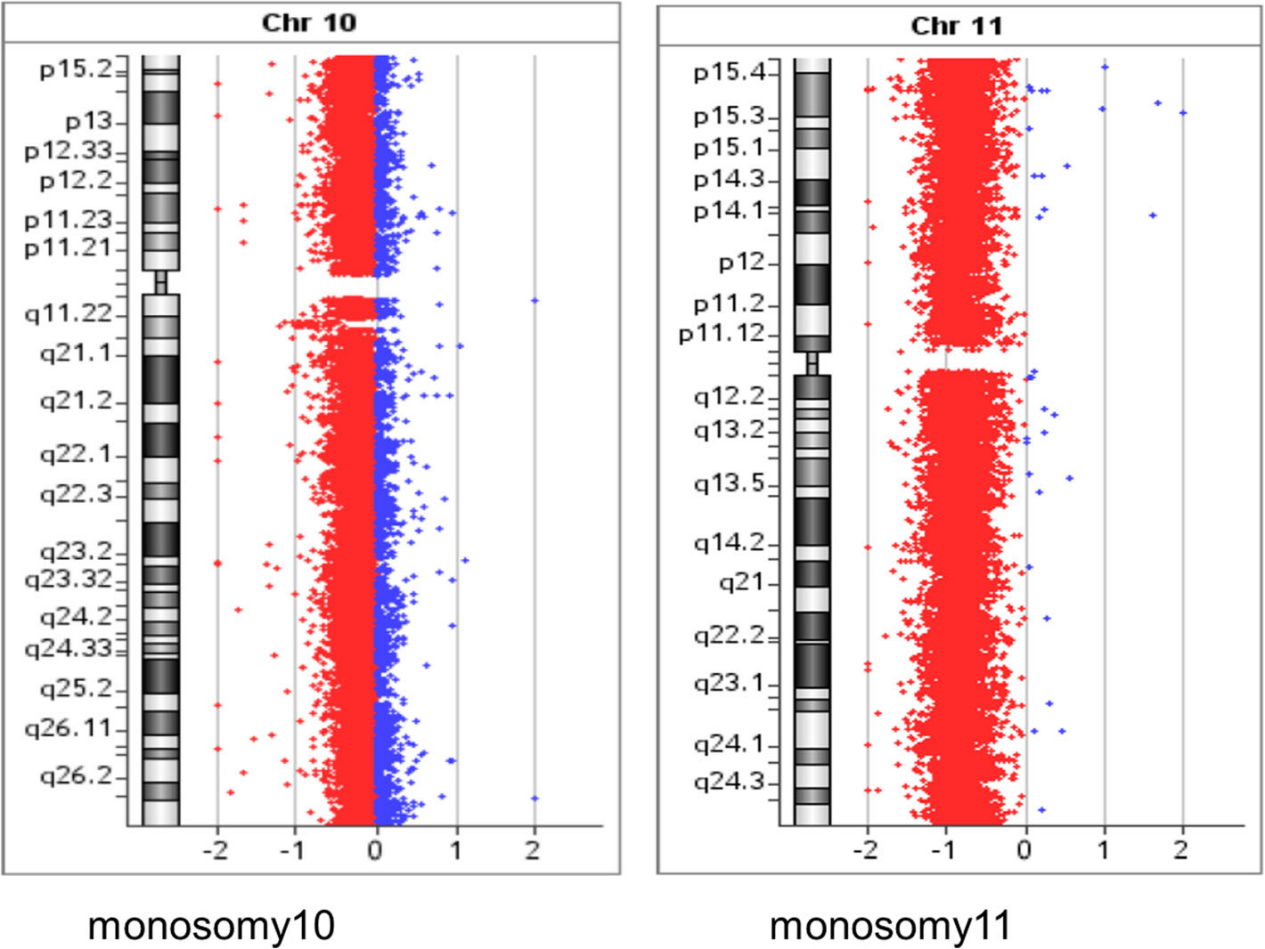
CGH array analysis showing loss of complete chromosomes 10 and 11 (red bar)

**Fig. 9:**
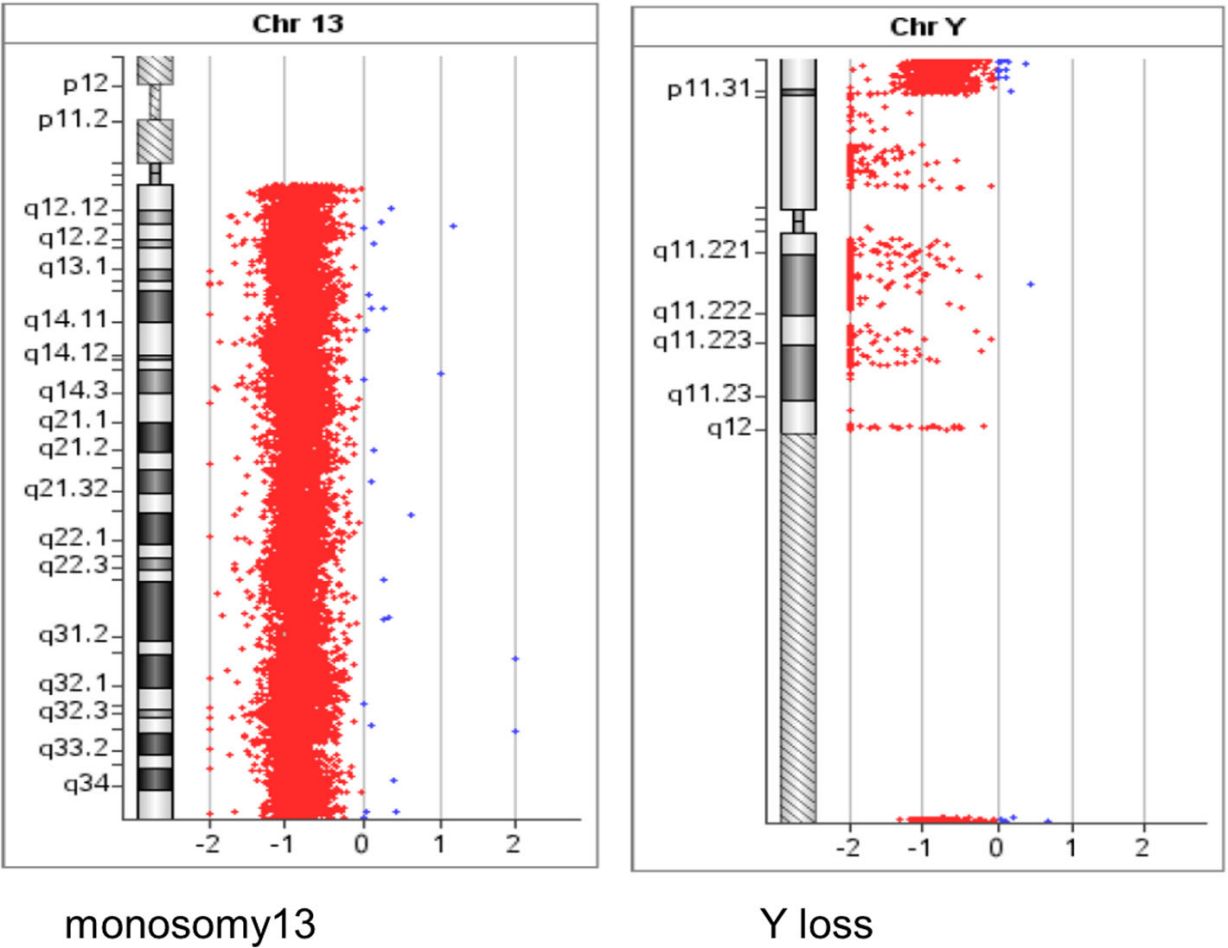
CGH array analysis showing loss of complete chromosomes 13 and Y (red bar)

To confirm the findings of the routine G-banded chromosomal analysis, we performed array CGH. Through this, we were able to determine the chromosomal origin of the observed DMs, and to detect possible submicroscopic chromosomal imbalances in this cell line. The array CGH results showed loss of complete chromosomes 10, 11, 13, 17, 18, and Y (Figs. 3, 4, Figs. 7, 8, 9). Moreover, the observed partial gain of chromosome 7 at q22.1, and partial loss of chromosome 1 at q32.1 confirmed the existence of unbalanced translocation between chromosomes 1 and 7 (Fig. 2c). Additionally, the loss of the entire q and p arms of chromosomes 16 and 17, respectively, further confirmed the presence of derivative chromosomes, during karyotype analysis (Fig. 3c). Interestingly, a high-level gain was detected in the 8q24 region, which corresponds to the MYC gene, and is likely to be attributed to the DMs observed during karyotype analysis (Fig. 4c).

Based on G-banding, array CGH and FISH analyses, this study has revealed the cytogenetic profile of the SK-PN-DW cell line. The important highlights of these findings are as follows: a 50.4-Mb terminal deletion on the distal chromosome 1q (del [1](q32.1qter)); a 43.66-Mb terminal deletion on the distal chromosome 16q (del [13](q11.2qter)); and a 22.15-Mb terminal deletion on the distal chromosome 17p (del [15](p11.1pter)). In parallel, some gains were also observed in this cell line, including: a 59.5-Mb terminal duplication on the distal chromosome 7q (dup [7](q22.1qter)) and an 8.2-Mb duplication on the distal chromosome 8q (dup [8](q24.23-q24.3)). Additionally, some contradictory results were also observed, for example, through array CGH analysis chromosome 22 was found to be normal, while karyotype and FISH analysis indicated the existence of a translocation between chromosome 11 and 22 (der [11], t(11;22)(11q;22q)). Interestingly, some novel translocations were detected among these rearrangements. For example, our analysis indicated monosomy at chromosomes 11, 13, and 18. (Table 2).

**Table 2:** Summary of the specific chromosomal rearrangement as detected in the SK-PN-DW cell line

Chr	Cytogenetic band	Position(Mb)	Mb	Gain/loss	1979y	1987y
X	P22.33	61,091-2,698,172	2.63	L	x	x
Y	-Y			L	y	y
1	q32.1q44	198,816,259-249,218,792	50.40	L		
7	7q22.1q36.3	99,622,633-159,118,566	59.50	G		
8	q24.21	128,465,623-129,012,209	0.55	G		
8	q24.23q24.3	138,096,903-146,294,098	8.20	G		
9		normal		N	−9	-9
10	−10			L	−10	−10
11	−11			L	−11	−11
12		normal		N		−12
13	−13			L	−13	−13
16	q12.1q24.3	46,500,741-90,163,114	43.66	L		
17	p13.3p11.1	51,885-22,205,821	22.15	L	iso(17q)	−17
17	q11.1q11.2	25,390,193-30,127,940	4.74	L		
18	−18			L	− 18	− 18

These results have been communicated by a poster in the conference of ASHG(2015).(http://www.ashg.org/2015meeting/pdf/57715_Posters.pdf?)

## Discussion

In the case of the der [1], t(1;7)(1q;7q) rearrangement, this was not initially observed through Karyotype analysis. However, the array CGH analysis did indicated the deletion of 1q32.1qter and the duplication of 7q22.1qter. This translocation was also confirmed through FISH analysis. Screening of the literature revealed that hundreds of oncogenes and tumor suppressor genes are found in these 1q loss and 7q gain regions. For example, Novel Ras Effector 1 (NORE1) is a gene that is localized on 1q32.1, and NORE1 and RASSF1A form homo and hetero dimers by associating with Ras-like GTPases, which may be important for its function as a suppressor gene of PNET [12, 16, 17].

The rearrangement of der [13], t(16;17)(16q;17q) is another translocation that was observed in our study. Initially, 18 cells (18/20, 90%) indicated monosomy at chromosome 17. However, after comparing the array CGH and FISH images, it was concluded that monosomy of chromosome 17 was the wrong conclusion. The suspected monosomy at chromosome 17 was instead identified as a translocation between chromosome 16 and 17. It was observed that the short arm 16q and the long arm 17p were deleted, and the long arm of 17q was translocated to the 16q location. This was very interesting observation. A study by Yin and colleagues also identified a loss of 16q and 17p [18]. Earlier studies investigating PNET also observed that the most common chromosomal abnormality observed is on chromosome 17q, while 17p is lost, indicating the presence of important tumor suppressor genes on 17p [13–15]. Consistent with these findings, our array CGH also identified the breakpoint of 17p at 17p11.1. It is evident that several tumor suppressors, including p53, are located within the deleted region of 17p13.1 [19]. Another independent study also indicated that the loss of 17p correlated with poor survival [20]. It should be noted that the loss of 16q is quite common in PNET, thus it would be reasonable to hypothesize that 16q loss might be associated with poor patient survival. It is highly possible that one or more suppressor genes that are located on 16q might play a vital role in pathology, and would be interesting to follow in future studies.

The third translocation that was observed was between chromosome 8 and 18. This rearrangement had not been previously reported. Based on the karyotype image analysis, monosomy at chromosome 18 was observed, and chromosome 8 appeared normal. However, the array CGH analysis showed two duplications, at 8q24.21(size, 547 kb) and 8q24.23–24.3 (size, 8197 kb), respectively. Following analysis of array CGH and FISH results, we finally concluded the existence of a novel rearrangement, del [14], t(8;18)(8q24.23q23;18pter). It seems that some oncogenes, including the myc family genes (MYC, MYCN, and MYCL1), were located on the 8q24 region of the chromosome [21–23]. These genes played an important role in tumor progression. In our FISH analysis, we used CEP8 and C-MYC probes to identify these important genes. Interestingly, it was observed that the myc gene was not only located on chromosome 8, but was also observed in the DMs. Additionally, our study demonstrated the presence of different quantities of DMs in each cell (range is 4–60 per cell), which reasonably indicate that myc genes are closely associated with tumor occurrence. In an independent study, amplifications of the myc family members has been identified in 5 to 15% of the patients that showed an association with a poor response to therapy [21, 22]. Similarly, the study by Roussel and Robinson separately accounted for the roles of the myc family genes in Medulloblastoma [24]. The amplification of the myc gene in PNETs has also been described previously [23, 25]. Another tumor suppressor gene, deleted in colorectal carcinoma gene (DCC), which has been shown to play an important role in mediating cell differentiation in the nervous system along with apoptotic processes was mapped onto chromosome 18q21.1 [26, 27]. However, more detailed analysis of this gene is required in the nervous system tumors of children.

Finally, another translocation was observed between chromosomes 11 and 22. Both array CGH and karyptype analyses indicated contrasting information regarding chromosome 22. The array CGH results indicated that chromosome 22 was normal, while Karyotype analysis demonstrated abnormal chromosome 11 and 22. The FISH analysis indicated the following rearrangement, t(11;22)(11q24;22q12). Recently, multiple studies have reported a role of this translocation in Ewing Sarcoma [28, 29]. The fusion gene EWSR1 was located at chromosome 22q12, and FLI1 was located at 11 g24 [30–32]. These translocations have the potential to impact p53 function by regulating multiple pathways [30–32].

Additionally, we also observed partial monosomy of chromosome 10, with the tumor suppressor gene, DMBT1, located at 10q25.3–26.1 [33, 34]. The PTEN gene, located at 10q23, has recently been implicated as a candidate tumor suppressor gene in brain, breast, and prostate tumors. Interestingly, the single most common change observed in all of the PNET cells was the loss of chromosome 13. In terms of its role in tumor pathogenesis, we do not have sufficient information. Our analysis of the SK-PN-DW cell line found many differences than previous analyses (Table 1) [9].

## Conclusion

Overall, our study concluded that the continuous culturing of cell lines induces changes in the copy number, and possibly affecting the function of many chromosomes, thus making them unstable and less authentic. Moreover, the authentication of these cell lines using individual analyses, such as karyotyping, array CGH, or FISH alone is not sufficient, as these analyses can yield varying results. Thus, a combination of these techniques should be used for authentication for important research. We analyzed only one cell line (SK-PN-DW) of PNET. Next stage we will continue to analyze the different generation of this cell line and other cell lines using the same methods, and showing more data.

## Abbreviations

PNET: malignant primitive neuroectodermal tumor CGH: Comparative genomic hybridization FISH Fluorescence in situ hybridization
